# Analysis of Current Status and Strategies of Retinopathy of Prematurity Screening during 6 Years in Local Regions of China: Implication and Caution

**DOI:** 10.1155/2014/756059

**Published:** 2014-11-04

**Authors:** Lu Chen, Ming Su, Sheng-gang Ren, Hui-lan Hua, Jian-cang Wang, Wei Zheng

**Affiliations:** Department of Ophthalmology, Children's Hospital of Hebei Province, No. 133, South Jianhua Street, Shijiazhuang 050031, China

## Abstract

*Purpose*. To understand the current status of retinopathy of prematurity (ROP) screening in a province of North China. *Methods*. We retrospectively analyzed 5651 cases with ROP screening in the Provincial Screening Center of Hebei Province from January 2008 to December 2013. *Results*. 14.98% of all ROP patients and 1.56% of severe ROP patients required treatment. All the severe ROP patients met the criteria of screening. Severe ROP patients were detected at recommended initial screening time (4–6 weeks after birth). The frequency of other ocular diseases was 8.03%, in which the main disease was fundus hemorrhage. In 2665 more mature and unqualified infants, only 2 retinoblastoma and 2 familial exudative vitreoretinopathy were detected, which indicates the advantage of early diagnosis and treatment based on fundus examination. *Conclusions*. It is suggested that the standard of GA < 32 weeks and/or BW < 1800 g could be served as the screening criteria in the local region for ROP screening. 4 weeks after birth is the most appropriate time for initial screening.

## 1. Introduction

Retinopathy of prematurity (ROP) is a proliferative retinal vascular disease in infants with a preference of either premature or low birth weight individuals. According to the statistics from World Health Organization (WHO), ROP is not only the leading cause of blindness in developed countries but also the great challenge of treatment in developing countries [1]. Following the initial attack to developed countries, developing countries are suffering from the third impacts of ROP [2]. As one of the largest developing countries, China has drawn significant attention from all over the world with the rapid development in economy, society, culture, and medical care. Consequently, the threats of ROP and ROP-related disorders and disability in movements, language, and psychology cause more and more concerns and research interests in the country.

The guidance of clinical work in baby-caring, diagnosis, and treatment for premature infants reduces the incidence of ROP. Ministry of Health of China issued the guidelines on the policies of oxygen usage and prevention and treatment of ROP in April 2004. Compared with the developed countries, ROP patients in China have their specific characteristics, such as larger ranges in gestational age (GA) and birth weight (BW). More importantly, due to the complexity of national composition and geographical difference and the variance in economy and culture across regions, it is a great challenge to make general criteria for ROP screening, which requires more focuses and specific studies based on large populations.

In the present report, we aimed to study the current status of ROP screening in Hebei province, a province in North of China. We retrospectively analyzed the medical records of 5651 infants with ROP examinations in the past 6 years in Hebei Provincial Screening Center. We also validated the current screening criteria, which could help guiding clinic work and avoiding ROP-related incidents of blind.

## 2. Materials and Method

### 2.1. Objectives

The medical records of consecutive infants admitted to the ROP screening center in Hebei province from 01-Jan-2008 to 31-Dec-2013 were retrospectively studied. The follow-up data were updated until 28-Feb-2014. All the infants were referred to our center by the doctors from various levels of other hospitals in Hebei province. This study was approved by the ethics committee of Children's Hospital of Hebei Province.

### 2.2. ROP Screening Strategy Recommended by Chinese Medical Association (CMA)

The suggested screening criteria by CMA are GA < 32 weeks and/or BW < 2000 g. CMA also recommends that infants who do not meet the criteria but with poor general conditions should also be included. It should be noted that the basis of these criteria is not very solid. Region-specific criteria are needed to be further evaluated and revised if necessary [3]. In this study, all examined infants are referred to our center for ROP screening. There are some premature and mature infants in our series. However, in order to verify the accuracy and feasibility of current screening criteria, we screened all the referred infants.

*Screening Procedure*. Ophthalmological examinations were performed for infants 4–6 weeks after birth. The results were recorded according to the revised international classification of ROP [4]. Time of subsequent follow-up examination was determined according to the fundus status, except for the cases who needed urgent treatment of severe diseases or whose retinae were with full vascularization in both eyes.

*Treating Criteria*. Only objectives with the type 1 ROP based on ET-ROP studies were treated, including zone I, any stage ROP with plus disease, zone I, stage 3 ROP with or without plus disease, and zone II, stage 2 or 3 ROP with plus disease [5, 6]. In addition, objectives with zone II or III, stage 3 with no trends of regression, were also included in the present study. We use the term “severe ROP” for all the diseases mentioned above, whereas other ROP cases were classified as “mild ROP.”

*Criteria of Stopping Screening*. (1) Complete retinal vascularization; (2) postmenstrual age (PMA) of 45 weeks and no severe ROP; (3) zone III vascularization, no lesions above stage 1, and without zone I or II ROP during the previous regular examinations; (4) two consecutive independent observations of regression of ROP. For the infant with mild ROP and who met the stop-screening criteria, a decision was made by the parents or guardians about whether or not continuing the follow-up examination to observe the development of retinal vessels.

### 2.3. Examination Methods

Parental or guardian informed consent was sought prior to ophthalmic examination and completion of charts. Approximately 90% of fundus examinations were performed by the authors of this work, while the other 10% were done by peer colleagues. The whole examination procedure includes eyes dilating, topical anesthesia, and eyelid opening with lid speculum and collection of fundus images with a wide-angle digital fundus camera (RetCam II, Clarity Medical Systems, Inc., Pleasanton, California, USA).

### 2.4. Statistics

Statistical analyses were performed using SPSS version 18.0 (SPSS Inc., Chicago, Illinois, USA). Categorical data were expressed as raw data and percentage. Chi-square test was used to examine the relation between categorical variables. Numerical data were presented as mean ± standard deviation (SD) and were compared using *t* test or one-way ANOVA where applicable. Differences were considered statistically significant when *P* < 0.05.

## 3. Results

### 3.1. Summary of Screening Objectives

**Figure 1:**
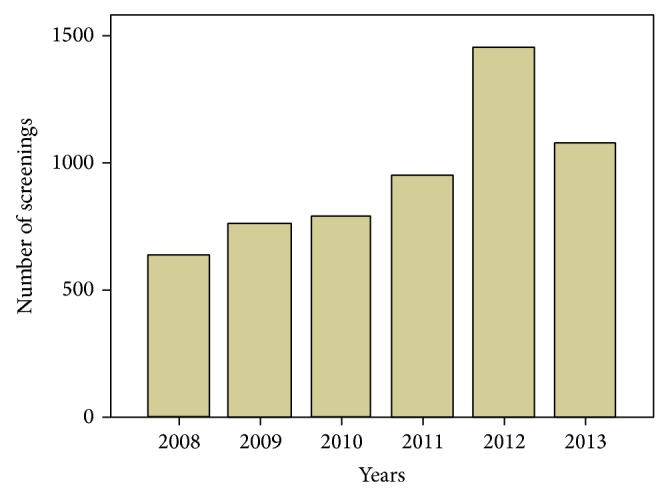
The changes of population in the screening study during the past 6 years.

As shown in Figure 1, the screening population consists of a total of 5651 infants. The number of cases per year increased during the past 6 years with a peak of 1452 enrollments at 2012, which increased by 53.65% compared with the number at previous year.

**Figure 2:**
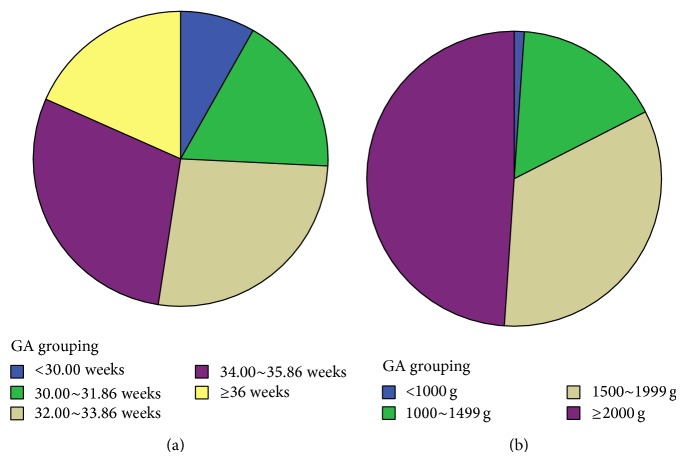
The distribution of infants with various gestational age and birth weights.

The data of GA and BW are summarized in Figure 2. It is shown that the mean GA was 33.49 ± 2.64 weeks (range 24–42.86 weeks), and the mean BW was 2000.14 ± 573.45 g (range 600–7200 g). There were 458 infants (8.12%) with GA < 30 weeks, 1000 infants (17.72%) with GA < 32 weeks, and 1036 infants (18.36%) with GA ≥ 36 weeks. As to the BW, there were 59 infants (1.05%) with less than 1000 g, 926 infants (16.41%) with less than 1500 g, and 2759 infants (48.88%) with more than 2000 g. There was no significant difference between the results over the past 6 years (all *P* > 0.05).

As to the gender, there were 3580 male infants (63.43%), which was significantly (*P* < 0.001) more than the females (2064 infants, 36.57%). However, there was a large variance among different years and a consistent increase in the number of females (*P* = 0.016).

As to the delivery manner, there were 2532 infants (45.36%) who adopted vaginal delivery. 3050 infants (54.64%) adopted cesarean delivery, which was significantly (*P* < 0.001) higher than the former. The incidence of cesarean delivery increased constantly during these 6 years (*P* = 0.043).

There were 2,978 (52.77%) infants who met the screening criteria. The number of qualified objectives in the total enrolments significantly decreased by years during the past 6 years (*P* = 0.029).

### 3.2. Time of Initial Screening Examination

The PMA of first screening was 40.88 ± 4.92 weeks (range 29.71–128.00 weeks), and the postnatal age of first screening was 7.38 ± 4.53 weeks (range 0.29–92.43 weeks). As shown in Table 1, the postnatal age was increased while GA was decreased, and this correlation was statistically significant (*P* < 0.001). The incidence of first screening at 4–6 weeks after birth only accounted for 38.34% (out of 2165) infants, and 461 infants (8.16%) were examined within four weeks after birth. 727 infants (12.89%) were examined after the deadline of stopping screening (PMA 45 weeks).

There were 10 infants (0.46%) and 46 infants (1.53%) who were found with severe disease at first examination for the groups of on-time and delayed screening, respectively. Among them, there were 1 infant (0.05%) and 13 infants (0.43%), respectively, suffering from retinal detachment. There were significantly more infants with severe ROP in delayed-visit group than that in on-time-visit group (all *P* < 0.05).

### 3.3. Screening Adherence

There were 7 infants who failed to be examined due to the parents' rejection, which were listed in Table 2.

In addition, there was 1 infant within zone I stage 1 ROP plus disease in both eyes at PMA 35.29 weeks (case number 2497). His parents refused any forms of treatments. 73.05% infants did not follow up on schedule, and contacts of 32.62% infants were lost during the study. The mean frequency of examination for those lost-contact infants was 1.27 ± 0.66 times (range 1–6 times).

Results of the last examination of 5632 infants (except 19 cases with either refusal or practical difficulties) are summarized in Table 3. Among them, the results of last examination for the 236 lost-contact infants with mild ROP are listed in Table 4. Our results indicated that, firstly, infants (within zone II stage 2 or 3, or zone III stage 3 ROP) with no plus were at high risk and needed close observations; and secondly, infants who were younger at the last examination were at higher risk that their fundus might have more severe diseases in the near future.

### 3.4. Analysis of Screening Strategies

Results of analyzing the population based on different screening criteria are shown in Table 5. For example, 2978 infants met the Chinese screening criteria, in which 735 infants had ROP and 87 infants had severe ROP. Among the other 2665 unqualified infants, 103 infants had mild ROP, while none had severe ROP. Normal fundus was found in the rest 6 infants with elusive standards.

2165 infants were screened 4–6 weeks after birth according to the recommendation. Among them, 10 infants were found with severe ROP at first visit with the mean first screening time of 5.01 ± 0.60 weeks, including one with retinal detachment.

### 3.5. Other Ocular Findings Except for ROP

**Figure 3:**
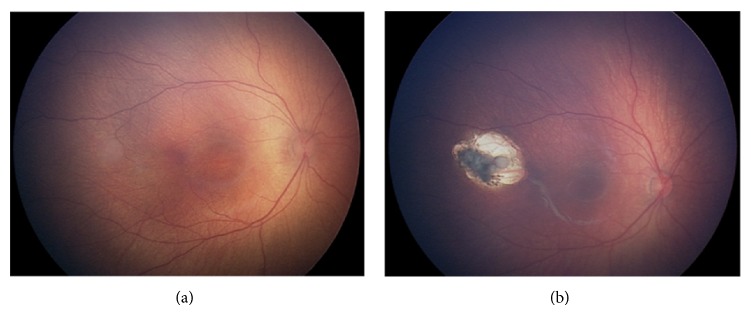
Comparison of both pre- and postsurgery in the case of RB in the right eye.

Of all objectives, other ocular lesions except for ROP were checked out in 454 infants (8.03%). And for those 2665 larger, more mature, and unqualified objectives, there were 187 (7.02%) infants checked out with other ocular lesions, including 93 retinal hemorrhage, 45 retinal pigment abnormalities, 20 congenital cataracts, 13 conjunctivitis or dacryocystitis, 7 optic nerve hypoplasia, 3 iris and/or choroid coloboma, 3 idiopathic congenital nystagmus, 2 familial exudative vitreoretinopathy (FEVR), 2 retinoblastoma (RB) (Figure 3), 2 persistent hyperplasia of primary vitreous (PHPV), 1 congenital glaucoma, 1 microphthalmus with leukoma, and 1 eyelid hemangioma.

## 4. Discussion

In the present study, it was shown that the majority of objectives were infants with relatively large GA and BW. The number of infants with BW < 1000 g and GA < 28 weeks is rather low. This trend was consistent in the past 6 years, as well as the ROP incidence. In addition, similar to a report by Gilbert et al. [1] about ROP in the developing countries, ROP or severe ROP was found in relatively mature infants. The studies by Courtright et al. [7] and Chen and Li [3] also suggested the characteristics for the screened population are much like the first epidemics. The reasons behind this could be various. However, it indicates the defects of current neonatal care services, especially for the premature infants, available in our local regions.

There were more than a half of cases adopting caesarean delivery in this study. Although it is highly related to the complicated conditions of pregnancy, the subjective factors also contribute considerably, such as parents' intended choice and staff members' compromise to the difficult practice environment, which in fact has constituted the leading cause of high incidence. Additionally, considering the potential increase in the acceptance of neonatal intensive care (NIC), caesarean delivery may serve as an indirect contribution to ROP incidence.

In the present study, some potential problems were noticed, such as too many objectives which failed to meet the screening criteria, the lack of standardization during screening procedure, and the poor adherence to the screening examination and treatment (not for too many times of examinations). All of these factors indicated the lack of understanding ROP as well as the faultiness in ROP screening and follow-up actions. These reasons delayed the detection and treatment of ROP, which resulted in blindness with the retinal detachment in the late term. Our findings highlighted the importance of awareness of ROP for both staff members and parents, which also suggested the need to strengthen the propaganda and education as well as fulfill the obligation. Fortunately, according to the records in our center, there was an increased incidence of screening recently due to the improved public awareness of ROP.

The safety, economy, and effectiveness of ROP screening require the strict criteria, which could eliminate the unnecessary inspection and largely reduce the waste of resource and efforts. From the results in Table 5, it is suggested that the cases in the present study were in accordance with the standards recommended by CMA. Missing of severe diseases could happen if we followed the standards in developed countries such as America and the United Kingdom, which is consistent with the opinion by Gilbert et al. [1]. Further analysis indicated that if ROP screening was limited to infants with GA < 32 weeks and/or BW < 1800 g, 21.16% or fewer infants would require screening compared with the present recommendation while missing no cases of ROP with severe disease. However, larger studies are definitely needed to achieve high confidence in the accuracy of revised criteria before it can be applied safely to clinical practice. Furthermore, it is particularly important to eliminate the negative social causes and poor practice environment in order to reduce the waste, which forces doctors to overrefer and overscreen in order to avoid medical disputes and prosecution.

Severe ROP and even retinal detachment were found during the initial examinations at recommended time (average time of 5.01 weeks). Therefore it is indicated that the time recommended by CMA is far from practical and useful, which can lead to missing the best time for treatment. Thus it is advised to adopt the protocol made by American Academy of Pediatrics [8], which revised the time for initial examination to 4 weeks after birth for the infants with a GA ≥ 27 weeks.

For the 2665 more mature infants, there were only 2 RB and 2 FEVR, which accounted both for 0.08%, respectively, and showed the advantage in early diagnosis and treatment based on fundus examination. Even for the rate of identifying RB, it was much more than the incidence of RB which could be partly attributed to the chance. Nie et al. [9] pointed out that there is obviously higher rate of identifying congenital diseases in neonatus accepted NIC than normal ones. Above all, it is suggested that we should popularize the universal newborn eye screening using reasonable program on one hand and provide more solid clinic validations for universal newborn fundus screening on the other hand. Now this suggestion has been proposed and implemented in some regions in China. After all, one screening program should not be brought out unless it is confirmed that the advantages outweigh the disadvantages. Our suggested criteria are in support of only the use of screening for ROP because there were only a very low proportion of patients who can be detected by fundus examination according to the fundus examination results for all newborns and mature newborns.

It is worth noting that our study varies from the previous reports by containing larger and more mature infants that failed to meet the screening criteria. Diseases such as FEVR, PHPV, Norrie's disease, and incontinentia pigmenti are all characterized by the similar clinic performances of ROP. FEVR was found in the premature infants [10], and there are numerous reports about older ROP patients in developing countries [11, 12]. It is always difficult to distinguish ROP from FEVR merely by referring to the short-term fundus observations, especially for the mild lesions. As a result, potential FEVR patients may exist in the present study.

There are some limitations in the study. First, due to difference in neonatal care services, GA calculation, and the uncompleted data, bias of potential resource should be considered. Second, there were only two ophthalmologists involved in this study, which might lead to observer bias. Above 90% workloads were completed by the authors in the present study, which minimized the bias. Finally, although the RetCam superior is beyond dispute, in agreement with other reports [13, 14], we consider image quality might be suboptimal for peripheral nasal zone II and peripheral zone III, particularly for Asian, which also might have led to diagnosis bias. Overall, with the limited practice environment in China, RetCam system is still the most reliable and convenient equipment for ROP screening.

## 5. Conclusions

GA < 32 weeks and/or BW < 1800 g can be used as the screening criteria in the local region to reduce the waste of resources. 4 weeks after birth is the most appropriate initial time for screening. Meanwhile, detection of severe ocular disease shows the importance of universal newborn eye screening with reasonable program, but the launch of universal newborn fundus screening must be careful.

## Figures and Tables

**Table 1:** The time of initial screening for infants with different gestational age.

Groups	GAs (weeks)	Postnatal age of initial screening (weeks)
Group 1 (<30.00 weeks)	28.62 ± 0.99	9.23 ± 4.73^∧^
Group 2 (30.00~31.86 weeks)	30.88 ± 0.61	8.07 ± 5.85^*^
Group 3 (32.00~33.86 weeks)	32.79 ± 0.61	7.10 ± 3.96^∗∧^
Group 4 (34.00~35.86 weeks)	34.75 ± 0.60	7.00 ± 4.22^∗∧^
Group 5 (≥36.00 weeks)	37.20 ± 1.39	6.91 ± 3.95^∗∧^
*F*		32.784
*P*		0.000

^*^Versus Group 1; ^∧^versus Group 2; GA = gestational age and it was expressed as mean ± standard deviation.

**Table 2:** The situation of infants that rejected to be examined by parents.

Medical record number	Year of screening	Gender	GA (weeks)	BW (g)	PMA (weeks)	History
2882	2010	Male	37.14	2600	46.86	—
3384	2011	Male	34.00	1850	46.57	HIE/CHD/intracranial hemorrhage/ventilator
3436	2011	Male	35.00	2900	40.29	Ventilator
3645	2011	Female	33.57	1960	38.29	Ventilator
4963	2012	Male	—	—	—	—
5094	2012	Male	35.57	2600	40.14	—
6002	2013	Female	34.43	1900	38.57	Intracranial hemorrhage

**Table 3:** Summary of results from the last examination.

	Number of cases	PMA of last examination (weeks)	Frequency of examination
Mild ROP	407	43.15 ± 6.21 (33.00~91.29)	2.03 ± 1.35 (1~7)
Severe ROP	88	40.64 ± 9.74 (32.00~112.57)	1.51 ± 0.98 (1~6)
Degenerative/scar ROP	52	58.46 ± 13.07 (39.00 ± 106.43)	3.46 ± 1.72 (1~9)
Not vascularization	329	36.86 ± 2.14 (31.43~49.57)	1.08 ± 0.30 (1~3)
Normal	4703	42.74 ± 5.78 (34.14~176.00)	1.27 ± 0.70 (1~9)
Unclear observation in peripheral fundus	51	38.82 ± 2.74 (34.14~44.86)	1.08 ± 0.27 (1~2)
FEVR (misdiagnosed as ROP at first)	2	96.36 ± 66.97 (49.00~143.71)	1.50 ± 0.71 (1~2)

ROP, retinopathy of prematurity; FEVR, familial exudative vitreoretinopathy; PMA, postmenstrual age.

**Table 4:** Summary of the last results from the lost infants with mild ROP.

Zone	Stage	Plus disease	Number of infants
2	3	Preplus	1
2	3	(—)	1
2	2	Preplus	4
2	2	(—)	8
2	1	(—)	2
3	3	Preplus	1
3	3	(—)	4
3	2	(—)	74
3	1	(—)	140
3	Degenerative	(—)	1

**Table 5:** Summary of analysis of ROP according to different criteria.

	Total member (*n*)	ROP (*n*1)	Severe ROP (*n*2)	Missing member (*n*3)
Chinese criteria^*^				
(1) Qualified	2978	735 (24.68%)	87 (2.92%)	43 (1.44%)
(2) Unqualified	2665	101 (3.79%)	0	25 (0.94%)
(3) Unclear	8	0	0	2 (25%)
American criteria^∧^				
(1) Qualified	1375	495 (36.00%)	77 (5.60%)	21 (1.53%)
(2) Unqualified	4265	341 (8.00%)	10 (0.23%)	47 (1.10%)
(3) Unclear	11	0	0	2 (18.18%)
British criteria^#^				
(1) Qualified	1351	508 (37.60%)	76 (5.63%)	23 (1.70%)
(2) Unqualified	4292	328 (7.64%)	11 (0.26%)	45 (1.05%)
(3) Unclear	8	0	0	2 (25%)
Proposed criteria^**^				
(1) Qualified	2348	667 (28.41%)	87 (3.71%)	39 (1.66%)
(2) Unqualified	3293	169 (5.13%)	0	29 (0.88%)
(3) Unclear	10	0	0	2 (20%)

^*^GA <32 weeks and/or BW <2000 g; ^∧^GA ≤30 weeks or BW ≤1500 g; ^#^GA <31 weeks and/or BW <1500 g; ^**^GA <32 w and/or BW <1800 g.

## Abbreviations

GA: Gestational age
BW: Birth weight
ROP: Retinopathy of prematurity
FEVR: Familial exudative vitreoretinopathy
PMA: Postmenstrual age
RB: Retinoblastoma.
